# Toxicity and Biotransformation of Carbon-Based Nanomaterials in Marine Microalgae *Heterosigma akashiwo*

**DOI:** 10.3390/ijms241210020

**Published:** 2023-06-12

**Authors:** Konstantin Pikula, Seyed Ali Johari, Ralph Santos-Oliveira, Kirill Golokhvast

**Affiliations:** 1 Polytechnical Institute, Far Eastern Federal University, 10 Ajax Bay, Russky Island, 690922 Vladivostok, Russia; pikula_ks@dvfu.ru; 2 Department of Fisheries, Faculty of Natural Resources, University of Kurdistan, Pasdaran St, Sanandaj 66177-15175, Iran; sajohari@gmail.com; 3 Laboratory of Nanoradiopharmaceuticals and Synthesis of Novel Radiopharmaceuticals, Nuclear Engineering Institute, Brazilian Nuclear Energy Commission, Rua Hélio de Almeida 75, Rio de Janeiro 21941906, Brazil; roliveira@ien.gov.br; 4 Laboratory of Nanoradiopharmaceuticals and Radiopharmacy, Rio de Janeiro State University, R. São Francisco Xavier, 524, Rio de Janeiro 23070200, Brazil; 5 Siberian Federal Scientific Center of Agrobiotechnology RAS, Centralnaya Str., Presidium, 633501 Krasnoobsk, Russia

**Keywords:** carbon nanotubes, graphene, fullerene, nanotoxicology, bioassay, growth rate inhibition, flow cytometry, nanoparticles, oxidative stress, environmental fate

## Abstract

This work is related to the environmental toxicology risk assessment and evaluation of the possible transformation of carbon-based nanomaterials (CNMs) after contact with marine microalgae. The materials used in the study represent common and widely applied multi-walled carbon nanotubes (CNTs), fullerene (C_60_), graphene (Gr), and graphene oxide (GrO). The toxicity was evaluated as growth rate inhibition, esterase activity, membrane potential, and reactive oxygen species generation changes. The measurement was performed with flow cytometry after 3, 24, 96 h, and 7 days. The biotransformation of nanomaterials was evaluated after 7 days of microalgae cultivation with CNMs by FTIR and Raman spectroscopy. The calculated toxic level (EC_50_ in mg/L, 96 h) of used CNMs reduced in the following order: CNTs (18.98) > GrO (76.77) > Gr (159.40) > C_60_ (414.0). Oxidative stress and membrane depolarization were the main toxic action of CNTs and GrO. At the same time, Gr and C_60_ decreased the toxic action with time and had no negative impact on microalgae after 7 days of exposure even at the concentration of 125 mg/L. Moreover, C_60_ and Gr after 7 days of contact with microalgae cells obtained structural deformations.

## 1. Introduction

For two decades, carbon-based nanomaterials (CNMs) have attracted the interest of the industry and scientific community [1,2,3], which is associated with a rapid growth of their production [4]. In 2021, the global CNM market was valued at USD 2.9 billion and is projected to reach USD 31.6 billion by 2031, according to a report by Allied Market Research (https://www.alliedmarketresearch.com/carbon-nano-materials-market (accessed on 5 May 2023)). Among the main and common representatives of CNMs’ family, we can highlight fullerene (C_60_ or C_70_), graphene (Gr), graphene oxide (GrO), carbon nanotubes (CNTs), carbon dots, and other derivatives, which found a wide range of application due to their unique mechanical, electrical, thermal, optical, and chemical properties [1,5,6]. Currently, CNMs are used as drug carriers [7,8], antibacterial agents [9], bio-sensors [10], compounds for bionanocomposites [11,12], energy conversion and storage devices [13,14], substrates for water purification [15,16], and metals recovery [17,18], etc.

Along with the massive growth of CNMs’ production, there is a serious concern related to the toxicity and possible environmental risks caused by the interaction of CNMs with living organisms [19,20,21]. Much of the current literature is related to the potential entrance of CNMs into aquatic systems with sewage, surface wash, atmospheric sedimentation, leakage, and other direct and indirect pathways [19,22,23]. The main registered source of these materials in aquatic environments is the application of CNMs for water purification [24,25,26]. Fullerene C_60_ has been detected in water from wastewater treatment plants in concentrations about the range of ng/L [19,27]. In 2009, Gottschalk et al. calculated predicted environmental concentrations of C_60_ and CNTs in the surface water of Europe and the USA [28]. The calculated values of CNTs were 0.004 and 0.001 ng/L for Europe and the USA, respectively. The calculated values of C_60_ were 0.017 and 0.003 ng/L for Europe and the USA, respectively. After five years, the same predictive model revealed 6 and 57 times higher environmental concentrations of C_60_ and CNTs in Europe, respectively [29]. This increase in the predicted concentrations was mainly caused by the larger production of CNMs. The predicted environmental concentration of graphene and graphene-family nanomaterials was obtained for the first time by Hong et al. (2022) using Dynamic and Probabilistic Material Flow Analysis [30]. According to this model, the predicted concentration of graphene-family nanomaterials in Europe would reach 1.4 ng/L in surface waters by 2030, and the increase therefore will be more than 1000-fold between 2010 and 2030. It should be highlighted that the existing predictive models did not cover the accidental release of nanomaterials (NMs), which could lead to significantly higher concentration of NMs in nearby water bodies for a certain amount of time.

The vast majority of studies on the aquatic toxicity of CNMs have been performed with concentrations of several magnitudes higher than the reported predicted environmental concentrations [31,32,33]. However, these studies explore the levels of toxicity and toxic mechanisms of CNMs in different species and provide an important body of data for further assessment and predictions aimed at maintaining a safe environment. In general, the environmental risk assessment of NMs represent a very difficult task because the toxicity of NMs significantly varies depending on particle properties such as size, shape, functional groups, oxygen content, surface charges, hydrophobicity, and defect sites [20]. Further, the toxic impact can significantly vary between the used test organisms, exposure protocols, sample preparation protocols, colloidal status, and transformation of NPs under environmental conditions [34]. For the same reasons, it is difficult to compare the results of different studies.

Traditionally, the aquatic toxicity of NPs has been tested in bioassays with different model organisms, such as bacteria, microalgae, invertebrates, and fish [35,36,37]. Based on the literature, the sensitivity to CNMs varied between aquatic species, where the most sensitive group of organisms was algae, followed by crustaceans, fish, and bacteria [19].

Overall, microalgae represent a very useful and important model for NMs’ risk assessment considering their high sensitivity to CNMs and the fact that they are the main producers of organic matter in water bodies, and a basic trophic level, which gives rise to all water food chains [38]. Commonly, microalgae bioassays indicate the effects of pollutants on growth rate conditions, chlorophyll and protein content, DNA damage, oxidative stress, enzyme activity, membrane polarization, cell size changes, and other biochemical or morphological changes in the cells [39]. CNMs mostly affect microalgae cells through mechanical damage, accumulation in the cell membranes, and subsequent shading effect, which causes photosynthetic efficiency reduction and growth inhibition of microalgae [40]. Cruces et al. (2021) showed that both GrO and CNTs decreased the metabolic and photosynthetic activity of cyanobacteria *Microcystis aeruginosa* without oxidative stress or membrane damage, which was most likely caused by light shading and cell aggregation [41]. Our previous work with marine microalgae *Porphyridium purpureum* and four types of CNMs demonstrated that the toxic impact of CNMs depends on the surface properties of CNMs and the surface properties of microalgae cells [42]. It was revealed that exopolysaccharide coverage of *P. purpureum* cells facilitated high aggregation of hydrophobic CNMs with microalgae cells which resulted in higher toxicity.

The other important factor affecting the toxic properties of CNMs in aquatic systems is the environmental transformation of NMs [34,43,44]. In aquatic environments, NPs undergo physical (aggregation, agglomeration, sedimentation, and deposition), chemical (dissolution, photochemical reactions, oxidation, sulfidation, etc.), and biological (biodegradation and biotransformation) transformations [45]. The main physical transformations of CNMs in water media involve particle size, porosity changes, and interaction with natural organic matter and other particles dispersed in the medium [46]. The chemical transformation of CNMs is related to the surface reactions, which can change their surface properties [46]. The favorite sites for chemical transformation for CNMs are the edges of graphene sheets and the areas of defect or metal catalyst localization [47,48]. Biodegradation of CNMs occurs due to interaction with enzymes, organisms, and individual cells [49].

The particular interest of this study includes the interaction of aquatic species, namely microalgae, with different types of CNMs. The contact of aquatic species with CNMs is an interesting field of study considering the potential of aquatic species for pollution adaptation, environmental remediation, or pollutant transformation, which could result in both decreases or increase in toxicity. Moreover, the transfer of CNMs between aquatic food chains was previously reported for systems including bacteria, microalgae, crustaceans, and fish [50,51,52]. The bioavailability, uptake, and removal of 55% absorbed CNTs by green algae *Desmodesmus subspicatus* were demonstrated in the work of Rhiem et al. [53]. However, the biotransformation of CNMs by microalgae cells has not yet been investigated.

This work represents the evaluation of toxic levels and toxic effects caused by the impact of four typical representatives of CNMs, namely multiwalled carbon nanotubes (CNTs), fullerene (C_60_), graphene powder (Gr), and graphene oxide (GrO) in marine microalgae *Heterosigma akashiwo*. This study also includes the evaluation of the possible transformation of the used CNMs after contact with microalgae cells. *H. akashiwo* was chosen as a widespread species [54], and as a common test object used in toxicity bioassays with different chemicals and substances, including NMs [55,56]. Moreover, our previous works demonstrated the toxic impact of two types of CNTs and two types of carbon nanofibers on *H. akashiwo*, confirming the suitability of the species for CNMs testing [55]. The used CNMs were chosen based on their representation among the group of carbon-based nanomaterials.

Although this work represents traditional in vitro bioassay with the used concentration of CNMs in several orders of magnitude higher than predicted and registered environmental concentrations, it provides the levels, mode of action, and transformation ability of four used types of CNMs after contact with marine microalgae *H. akashiwo*.

## 2. Results

### 2.1. Growth Rate Inhibition and Cell Size Changes

**Figure 1:**
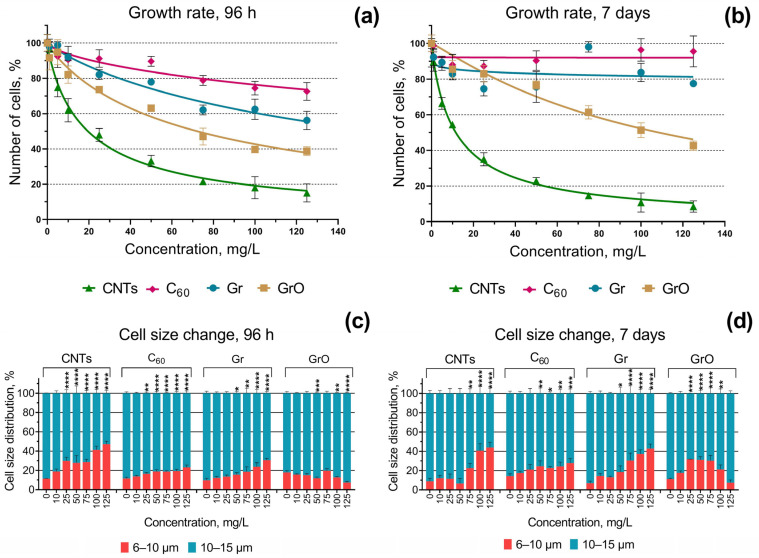
The growth rate inhibition and cell size distribution of *H. akashiwo* after the exposure to CNMs: (**a**) growth rate inhibition after 96 h of the exposure; (**b**) growth rate inhibition after 7 days of the exposure; (**c**) cell size distribution after 96 h of the exposure; (**d**) cell size distribution after 7 days of the exposure. *, *p* < 0.05; **, *p* < 0.01; ***, *p* < 0.001; ****, *p* < 0.0001. 95% confidence limits presented by the whiskers.

The no observed effect concentration (NOEC) and calculated effective concentrations of the four used CNMs which caused 10% (EC_10_) and 50% (EC_50_) inhibition of microalgal growth rate, as well as NOEC concentrations of microalgae cell size change, are represented in Table 1. The changes in the microalgae growth rate and cell size distribution after 96 h and 7 days of exposure to four types of CNMs are represented visually in Figure 1. The data of all the statistical significance calculations are represented in Table S1.

The growth rate inhibition results (Table 1, Figure 1a,b) demonstrated the highest toxicity of sample CNTs to microalgae *H. akashiwo*. All the tested samples can be listed from the higher to lower cytotoxic effect in the following order: CNTs > GrO > Gr > C_60_. All the samples had dose-dependent cytotoxicity in microalgae *H. akashiwo* after 96 h of exposure (Figure 1a). However, after 7 days of exposure, only sample CNTs showed an increase in toxic level, while the toxic impact of all the other samples on microalgae growth rate was reduced (Figure 1b). Furthermore, samples Gr and C_60_, which have the lowest toxicity among the others, after 7 days of exposure demonstrated almost no toxic impact on the growth rate of microalgae cells even at the highest used concentration, namely 125 mg/L (Figure 1b). At the same time, these samples caused significant growth rate inhibition after 7 days of exposure at concentrations of 5–25 mg/L (Table S1), which most likely means sedimentation of the particles at higher concentrations and microalgae adaptation to these conditions.

All the used CNMs had a significant impact on the size distribution of microalgae cells (Figure 1c,d). Samples CNTs, C_60_, and Gr caused a decrease in microalgae cell size after 96 h and 7 days, as significantly more cells appeared in the size range of 6–10 µm compared to the control. It should be noted that after 7 days of the exposure, the impact of CNTs on microalgae cell size distribution has reduced at concentrations below 100 mg\L, C_60_ caused the same cell size change in both time points, and Gr provoked higher microalgae cell size decrease after 7 days compared to 96 h exposure (Figure 1c,d). In contrast to all the other samples, GrO caused the increase in microalgae cell size after 96 h exposure (Figure 1c) at the highest concentrations, and more cells were registered in the size range of 10–15 µm. After 7 days of exposure, GrO caused a decrease in the size of *H. akashiwo* cells, the same way as demonstrated in all the other samples, but the effect was not dose-dependent and reduced at concentrations above 75 mg/L.

**Figure 2:**
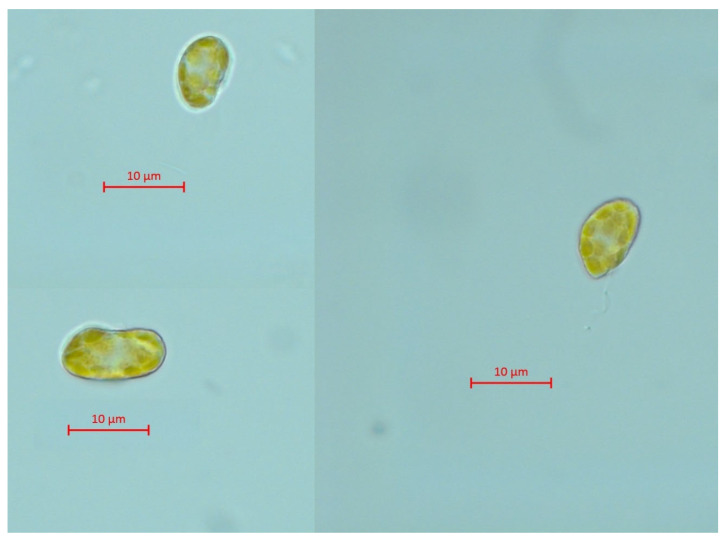
Microscopic pictures of *H. akashiwo* from the control group after 7 days of exposure. Magnification 1000×.

**Figure 3:**
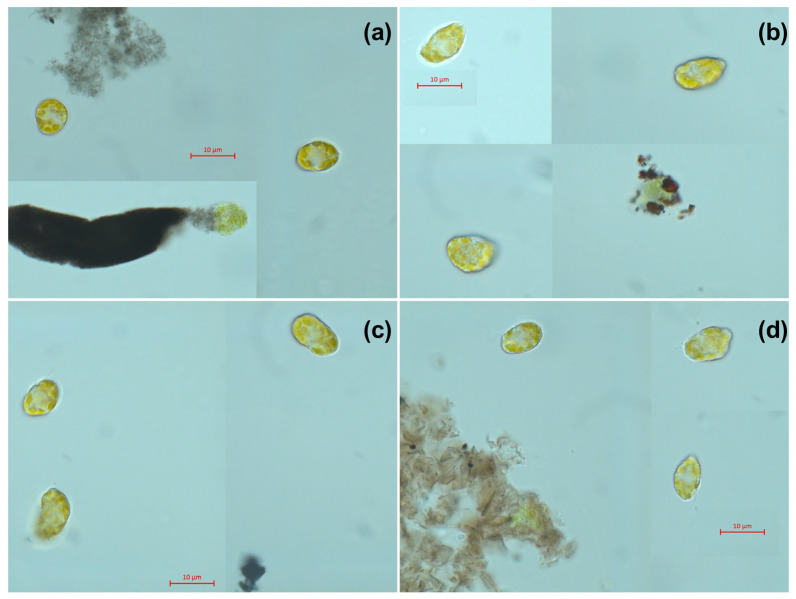
Microscopic pictures of *H. akashiwo cells* after 7 days of exposure to CNMs at a concentration of 25 mg/L: (**a**) multiwalled carbon nanotubes (sample CNTs) (**b**) fullerene (sample C_60_); (**c**) graphene powder (sample Gr); (**d**) graphene oxide (sample GrO). Magnification 1000×.

The microscopic pictures of *H. akashiwo* cells from the control group after 7 days of exposure are represented in Figure 2. Microalgae cells after 7 days of exposure to four types of CNMs are represented in Figure 3.

Microscopic observation demonstrated spherical-shaped cells of *H. akashiwo* exposed to CNTs (Figure 3a), and irregular, dissected form of the cells exposed to samples C_60_ and GrO (Figure 3b,d), compared to the relatively smooth shape of the cells of the control group (Figure 2). Moreover, cell debris was observed in agglomerated clusters of CNMs. At the same time, there were no alive microalgae cells absorbed with agglomerated CNMs. This observation can indicate the high sensitivity of shell-less *H. akashiwo* cells [57] to mechanical damage associated with cell-particle interaction.

### 2.2. Cellular Response Evaluation

**Figure 4:**
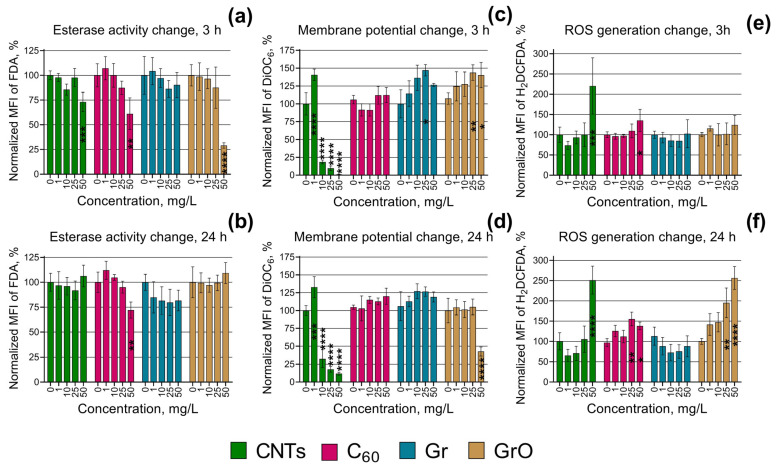
Biochemical changes in microalgae cells after 3 and 24 h of exposure to CNMs: (**a**,**b**) esterase activity change after 3 and 24 h, respectively; (**c**,**d**) membrane potential change after 3 and 24 h, respectively; (**e**,**f**) reactive oxygen species (ROS) generation change after 3 and 24 h, respectively. *, *p* < 0.05; **, *p* < 0.01; ***, *p* < 0.001; ****, *p* < 0.0001. The used endpoints were calculated compared to the control, where 0% is negative control (heat-treated cells) and 100% is positive control (cells with no exposure to nanoparticles).

The NOEC values of esterase activity, membrane potential, and ROS generation changes are given in Table 2. The changes of all these endpoints after the exposure of *H. akashiwo* to four types of CNMs are represented visually in Figure 4.

Figure 4a demonstrates that CNT, C_60_, and GrO had early responses after 3 h exposure to these CNMs with esterase activity inhibition. This effect was observed only for the highest concentration used in this series of assays (50 mg/L). Moreover, in the cases of CNTs and GrO, microalgae esterase activity was normalized after 24 h (Figure 4b). Sample Gr revealed no significant esterase activity change after both 3 and 24 h of the exposure. These results can indicate the adaptational ability of *H. akashiwo* cells to the impact of CNMs.

Considering the change in microalgae membrane potential, the highest membrane depolarization was registered after 3 and 24 h of CNTs exposure at concentrations 10–50 mg/L (Figure 4c,d), and after 24 h exposure to GrO at 50 mg/L (Figure 4d). Interestingly, CNTs concentration of 1 mg/L after 3 and 24 h exposure, Gr at 25 mg/L, and GrO at 25 and 50 mg/L after 3 h exposure caused hyperpolarization of cell membranes (Figure 4c). All the registered hyperpolarization effects neutralized after 24 h of the exposure (Figure 4d). Sample C_60_ with the lowest cytotoxic effect (Figure 1a,b) caused no significant effect on the membrane potential of microalgae cells (Figure 4c,d). Based on these results, we can highlight that in cases with high toxic impact (CNTs and high concentrations of GrO) microalgae cells undergo depolarization and followed by dysfunction and death, while low toxic impact (Gr at 25 mg/L, and low concentrations of GrO) provoked cell hyperpolarization which can result either in adaptation to the toxicant or in following cell exhaustion and membrane depolarization. Correspondingly, the samples with no significant toxic impact (C_60_ and Gr at all concentrations except 25 mg/L) caused no change in microalgae membrane polarization.

The highest ROS generation increase was observed for CNTs at 50 mg/L both after 3 and 24 h exposure (Figure 4e,f). GrO, the sample with the second microalgae cytotoxicity increased microalgae ROS generation at concentrations 25 and 50 mg/L only after 24 h exposure, and it demonstrated no effect after 3 h exposure. Interestingly a relatively moderate ROS generation increase caused the least toxic sample C_60_ (at 50 mg/L after 3 h, and at 25 and 50 mg/L after 24 h exposure). These results partly indicate the mode of action for the used CNMs and activation of microalgae adaptation mechanisms. In other words, the toxic action of CNTs and GrO includes oxidative stress, and in this case, *H. akashiwo* was unable to tolerate the level of ROS generation increase with the inner antioxidative system. However, this adaptation probably was successful for the lower oxidative stress impact, caused by the C_60_ sample, and the ROS generation increase did not result in microalgae growth rate inhibition.

### 2.3. Particle Biotransformation Assessment

**Figure 5:**
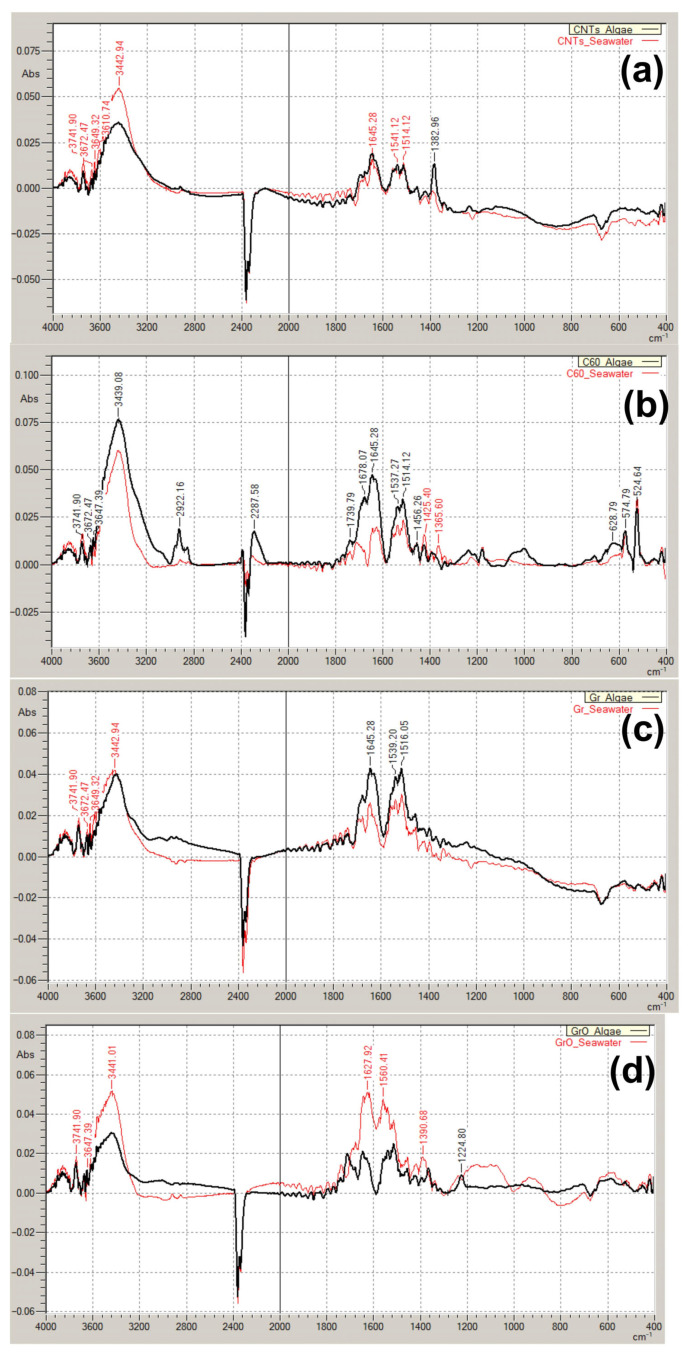
FTIR spectra of CNMs incubated in seawater with and without microalgae for seven days at a particle concentration of 25 mg/L: (**a**) CNTs; (**b**) C_60_; (**c**) Gr; (**d**) GrO. Red line, CNMs incubated in seawater without microalgae; black line, CNMs incubated in seawater with microalgae.

Fourier-transform infrared spectroscopy (FTIR) analysis was applied to assess the possible transformation of the tested CNMs after the incubation with microalgae cells for seven days. The comparison of FTIR spectra obtained from the particles incubated in only seawater and seawater with microalgae is represented in Figure 5. The used concentration of CNMs was 25 mg/L.

In general, it should be marked that all the obtained spectra have a specific broad absorption band at 3600–3200 cm^−1^, which is reported as a characteristic vibration mode for hydroxyl groups from intermolecular hydrogen-bonded OH:OH or absorbed water [58,59] and probably indicated the residual moisture in the samples. However, the spectra of pristine CNMs which had no incubation in seawater and dried overnight in the oven with the other samples had the same pick in this area (spectra not represented). This means that the sample preparation and measurement should be re-evaluated to exclude moisture absorption during FTIR measurement.

Compared to seawater control, sample CNTs obtained a pick of 1382.96 cm^−1^ (Figure 5a) which is typical of C-H bonds and reported for CH_2_ or CH_3_ [60].

Fullerene C_60_ after seven days of incubation with microalgae culture demonstrated the pick in the alicyclic area between 3000 and 2900 cm^−1^ (Figure 5b), which also reported for fullerene C_60_ as CH_2_ or CH_3_ impurities [61,62]. The absorption bands at about 1850–1750 cm^−1^ may be assigned to the oxidation of C_60_ [58,61,62], which has a more intensive signal after incubation with microalgae (Figure 5b). The bands of 1530–1460 cm^−1^ were previously reported for C_70_ fullerene [63]. The bands of 570–550 cm^−1^ indicated the coexistence of C_60_ and C_70_ fullerenes [64].

In Figure 5c, the absorption bands at 1645 and about 1545–1530 cm^−1^ indicate an amide group in graphene structure [65,66]. Graphene oxide (Figure 5d) indicated no observable modifications after the exposure to microalgae compared to seawater control, except the band at 1225 cm^−1^ which might be attributed to epoxy group modification of GrO [65,67].

**Figure 6:**
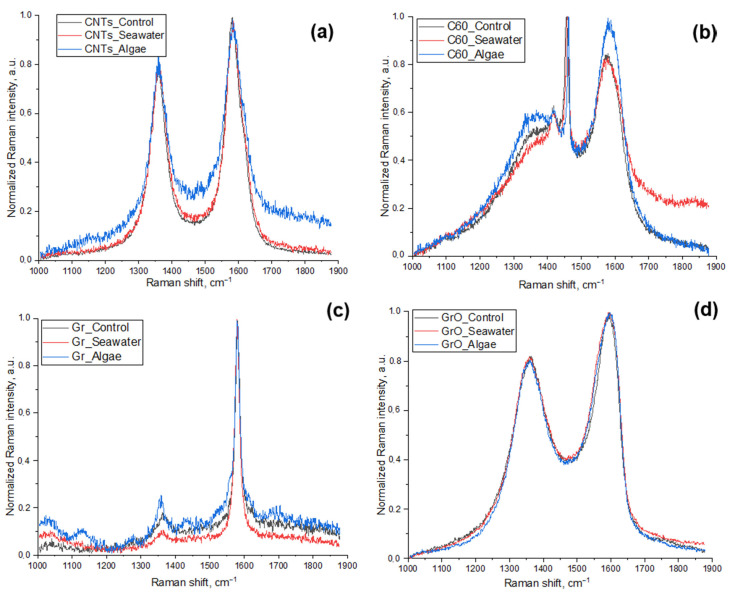
Raman spectra of CNMs incubated in seawater with and without microalgae for seven days at a particle concentration of 25 mg/L: (**a**) CNTs; (**b**) C_60_; (**c**) Gr; (**d**) GrO; Black line, initial CNMs without any exposure; red line, CNMs incubated in seawater without microalgae; blue line, CNMs incubated in seawater with microalgae.

The results of Raman spectroscopy analysis are represented in Figure 6. Except for the samples incubated in seawater, we also analyzed the initial CNMs without any exposure.

All the obtained spectra revealed the areas typical for CNMs, namely the D band (disorder-induced mode, around 1300 cm^−1^) and the G band (graphite mode, around 1550 cm^−1^). It should be noted that the best match of the spectra demonstrated sample GrO (Figure 5d), which means that after seven days of incubation in seawater with and without microalgae cells graphene oxide nanoparticles had no structural changes. CNTs also revealed a similar intensity both in the D and the G bands (Figure 6a) but had some signs of impurity in all the other areas of obtained spectra compare to pristine and seawater-incubated CNTs. C_60_ demonstrated an increase in intensity both around the G and the D bands compared to the same material not exposed to microalgae (Figure 6b), which indicates the potential of fullerene C_60_ to highest biotransformation among the other tested CNMs. Gr revealed an increase only in the D band (Figure 6c) in the probe, exposed with microalgae, which demonstrated the increase in structural disorder in graphene.

## 3. Discussion

Considering the growing interest in the production and application of different types of CNMs, the problem of their safety evaluation becomes more important. The present study was designed to determine the differences in the effect of multiwalled carbon nanotubes, fullerene, graphene powder, and graphene oxide in marine microalgae *H. akashiwo,* and to assess the possible biotransformation of these materials after the interaction with microalgae cells.

One of the most interesting results of this study is the demonstration of strong membrane depolarization in microalgae cells under the impact of the samples with the highest observed toxicity (Figure 4c,d), namely CNTs and GrO. The stable function of cellular membrane permeability and mitochondrial membrane potential represents a crucial component of the normal physiological function of cells [68]. Therefore, depolarization of microalgae membranes is a mark of upcoming cell death.

Moreover, the toxicity of CNTs and GrO was coupled with increased ROS generation in microalgae cells (Figure 4e,f). Intensive ROS formation causes destruction in proteins, lipids, and carbohydrates, and leads to oxidative stress in microalgae [69]. Except for mechanical impact and shading effect on microalgae, the toxic impact of CNTs might be associated with metal impurities remaining after the catalytic production of the particles [70]. The used sample of CNTs contains residuals of Al and Co (characteristics of the used CNMs are given in Section 4.1). It was reported that Co might become toxic at high concentrations, despite it being one of the essential metals for cell function [69,71]. Al can induce oxidative stress, ultrastructural changes, changes in lipid metabolism, degradation of cellular organelles, and suppression of antioxidant enzymatic activity in microalgae [72,73]. Probably, the presence of metal residuals caused oxidative stress in *H. akashiwo*, which further resulted in membrane dysfunction and cell death.

It is known that graphene family NMs could directly penetrate the cell membrane of algae through cell pores [74,75,76]. It was reported that GrO enters into the cells of *Chlorella vulgaris* and damage organelles, enhanced the generation of ROS, and disrupted antioxidant enzymes [77]. These results are in agreement with our study where GrO caused a 2.5-fold increase in ROS production in *H. akashiwo* cells after 7 days of exposure to a GrO concentration of 50 mg/L (Figure 4f).

Interestingly, *H. akashiwo* revealed a high adaptational ability to the impact of the used CNMs, except CNTs. It can be seen by a significant increase in EC_10_ and EC_50_ concentrations of microalgae growth rate inhibition after 7 days of exposure to GrO, compared to 96 h of exposure (Table 1). Moreover, samples C_60_ and Gr after 7 days of exposure had no toxic impact on the growth of *H. akashiwo* even at a concentration of 125 mg/L.

It is assumed that mechanical damage and oxidative stress are the main reasons for CNMs toxicity to *H. akashiwo* [76,78]. In this study, we can conclude that the shading effect had no strong impact on the growth of microalgae because of particle agglomeration and sedimentation, and the fact that *H. akashiwo* is motile and mostly surface living species [79]. Therefore, the cells of *H. akashiwo* could not be subjected to the prolonged mechanical impact of CNMs if they would rapidly be agglomerated and sedimented. In this study, the highest agglomeration and sedimentation rate in seawater had sample CNTs. The reasoning above also excludes the prolonged shading effect of sedimented CNMs. Hence, only the samples which caused oxidative stress had a significant toxic impact on *H. akashiwo*.

Despite there being no stated and widely accepted environmentally relevant concentrations of the graphene family and carbon nanomaterials, it can be estimated in the order of µg/L or even ng/L [80,81]. The calculated EC_10_ concentration for CNTs was between 1 and 2 mg/L and for GrO between 8 and 35 mg/L, based on the results of 96 h and 7 days of exposure (Table 1). This level of toxicity surpasses the possible environmentally relevant concentrations of CNMs by several orders of magnitude. However, it is important to establish the level of toxicity for different materials and consider their possible transformation and combined action with other toxicants in the environment.

Environmental transformation, such as agglomeration, interaction with natural organic matter (NOM), “biomolecular corona” formation, and interaction with organisms can change the bioavailability and toxicity of CNMs [82,83]. This study revealed that the most stable of the used CNMs was GrO, which showed no changes in FTIR and Raman spectra after the incubation with microalgae. CNTs obtained only CH_2_ and CH_3_ inclusions and have no structural defects. C_60_ obtained CH_2_ and CH_3_ inclusions, was oxygenated and achieved a more disordered structure. Gr undergo inclusion in the amide group and indicated structural disorder. Based on the results of the biotransformation study, it should be concluded that structural disorders were increased only in samples with the lowest toxicity (C_60_ and Gr). The contact of *H. akashiwo* with these types of CNMs was less dangerous to microalgae cells, which allowed them to have more interaction.

These findings suggest that long-term incubation of CNMs with microalgae could further increase the transformation of the NMs. However, this assumption is fair only for the NMs with low toxicity. Another interesting issue for further study is the toxicity testing of aged CNMs which had previously undergone biotransformation in contact with aquatic organisms. Moreover, future studies should implement more species from different trophic levels in the assessment of different common fictionized and pure CNMs, including the assessment of food chain transfer.

## 4. Materials and Methods

### 4.1. Nanoparticles

In this work, we used four types of CNMs (Table 3), namely multi-walled carbon nanotubes (CNTs), fullerene (C_60_), graphene powder (Gr), and graphene oxide (GrO). These types of NPs were chosen to compare the toxic effects and biotransformation between different CNMs in contact with marine microalgae *H. akashiwo*.

### 4.2. Microalgae Culture

The culture of a raphidophyte microalgae *Heterosigma akashiwo* MBRU_HAK17 (Raphidophyceae) [57] was provided by The Resource Collection “Marine biobank” (http://marbank.dvo.ru/index.php/ru/kollektsiya/microalgae/details/5/59 (accessed on 9 May 2023)) of the National Scientific Center of Marine Biology, Far Eastern Branch of the Russian Academy of Sciences (NSCMB FEB RAS). *H. akashiwo* is a useful object for bioassays and it is commonly used in ecotoxicology [84,85,86]. The morphology of *H. akashiwo* was described in detail [54,57]. It should be highlighted that the cells of *H. akashiwo* do not have a shell compared to most of the algae species, instead, the cells have amorphous vesicles under the cell wall [57]. *H. akashiwo* has a mixed feeding, which includes photosynthesis, direct nutrition uptake, and eating of the bacterium [87].

Culturing of microalgae and toxicity test conditions were maintained following the guidance of OECD No. 201 [88], with minor modifications as stated below. Microalgae were cultured with Guillard’s f/2 medium [89]. Filtered (pore diameter of the filter was 0.22 µm) and sterilized seawater with salinity 33 ± 1‰, pH 8.0 ± 0.2 was used for the experiments. The cultivation was carried out at a temperature of 20 ± 2 °C with an illumination intensity of 300 µmol photons/m^2^s, and a light:dark cycle of 12:12 h. All the bioassays were performed under the same conditions.

Before the experiment microalgae cells were cultivated in 250 mL Erlenmeyer’s flasks. Algal culture in the exponential growth phase was taken for bioassays. The initial cell density for all the bioassays was 1.5–2 × 10^4^ cells per mL.

### 4.3. Experimental Design and Sample Preparation

In general, all the performed experiments can be divided into three following parts: (1) microalgae growth inhibition assessment, (2) evaluation of cellular biochemical responses, and (3) the assessment of CNMs biotransformation by FTIR and Raman spectroscopy.

Before each series of experiments, the used CNMs were diluted into filtered and sterilized seawater (the same as used for microalgae culturing) to exclude the negative impact of salinity change on microalgae. The prepared stock concentration for all the used CNMs was 250 mg/mL. To prevent the agglomeration of NPs, the stock suspensions were sonicated with ultrasound homogenizer Bandelin Sonopuls GM 3100 (Bandelin Electronic GmbH & Co. KG, Berlin, Germany) with a high-frequency power of 100 W for 30 min. The sonication was performed on ice in 40 mL Sonopuls Rosette cell RZ-2 to prevent sample heating.

The first part of the study, namely the growth rate inhibition test, was performed in 24-well plates, where each well contained 1 mL of microalgae aliquots and 1 mL of the tested sample. The wells of the control group had only 1 mL of microalgae aliquots with the addition of 1 mL of f/2 medium. The treated wells had 1 mL of microalgae, and the other 1 mL of liquid included the calculated volume of the prepared CNMs stock suspension and f/2 medium to obtain the final exposure concentrations of 1, 5, 10, 25, 50, 75, 100, and 125 mg/mL. All the concentrations and control groups for each tested CNM were executed in quadruplicate. Cell count was performed with a flow cytometer CytoFLEX (Beckman Coulter, Indianapolis, IN, USA) after 96 h and 7 days of exposure. The protocol of cell count and post-processing of the results are described in the following section.

The second part of the study, which was the evaluation of cellular biochemical responses, included the assessment of esterase activity, membrane potential, and ROS generation changes in microalgae cells. These tests were performed separately in 24-well plates as described above, but the used final exposure concentrations were 1, 10, 25, and 50 mg/L. The concentrations were chosen based on the calculated in the previous stage EC_10_ and EC_50_ values. The measurements were executed after 3 and 24 h of the exposure to assess early response and its further dynamic. To assess the chosen biochemical responses, the sample from each well of 24-well plates was gently pipetted and 500 µL of liquid was transferred to a 48-well plate and stained with fluorescent dyes as described in the following section. All the used concentrations and control groups were executed in quadruplicate.

The third part of the study included the assessment of CNMs’ biotransformation. This experimental series was performed in 250 mL Erlenmeyer’s flasks. To evaluate the transformation of CNMs after the incubation with microalgae cells, all the used CNMs were incubated separately in filtered sterilized seawater (comparative control) and with microalgae aliquot (treatment). The only chosen concentration of CNMs was 25 mg/L. The total volume of liquid in each replicate was 100 mL, which included 50 mL of CNMs suspension and either 50 mL of seawater or 50 mL of microalgae aliquot. All the tests were performed in triplicate. The duration of exposure was seven days. Every day the flasks were gently shaken.

The method of CNMs separation for FTIR analysis was described in the work of Chouhan et al. [90]. After seven days, all the samples were collected and centrifuged in 50 mL centrifuge tubes with Eppendorf Centrifuge 5810R (Eppendorf, Hamburg, Germany) at 3900 rpm and 4 °C for 20 min. The pellets were washed once with deionized water obtained with Siemens Ultra-Clear TWF EDI UV UF TM Water Purification System (Siemens, Munich, Germany) and three times with methanol. To detach microalgae cells and cell debris, the samples were sonicated by ultrasound homogenizer with the high-frequency power of 40 W for 5 min. Then the samples were centrifuged at the same conditions as before, the supernatant was withdrawn, and CNMs from the pellets were phase separated in 1:1 mixture of hexane and deionized water. The separated NMs were collected from the thin ring on the phase-contact area and transferred to glass vials. The samples were dried in the oven overnight at 100 °C and stored in a desiccator until used for FTIR and Raman spectroscopy analysis. The samples with microalgae and the ones with only seawater were collected by identical procedure to exclude the impact of different separation protocols.

### 4.4. Flow Cytometry: Cell Count, Staining Protocols, and Post Processing

CytoFLEX flow cytometer (Beckman Coulter, Indianapolis, IN, USA) with the software package CytExpert v. 2.5 was used to measure the growth rate inhibition, size, and biochemical changes in microalgae cells after exposure to CNMs. The application of fluorescence dyes allowed us to distinguish live and dead microalgae cells and evaluate biochemical changes in exposed microalgae cells. All the used endpoints and parameters of their registration are summarized in Table 4. In all the cases, the excitation source was a blue laser (488 nm) of the CytoFLEX flow cytometer. The excitation source and emission channels were selected according to the maximum emission of the used fluorescent dyes, provided by the manufacturer (Molecular Probes, Eugene, OR, USA). The optimization of the used dye concentrations and duration of staining were described in our previous work [91] and re-evaluated directly before each series of measurements.

The evaluation of microalgae growth rate inhibition was completed with direct cell counting. Microalgae cells were distinguished using the parameters of microalgae cell size and granularity (in dot cytogram of forward and side scattering of blue laser), and fluorescence of chlorophyll *a* (emission channel 690 nm). To exclude dead cells from the count, the probes were stained with propidium iodide (PI) according to the standard bioassay protocol [92]. The mechanism of PI action is the incorporation between DNA or RNA base pairs, whereupon the dye increases its fluorescence intensity by 20–30 times [93]. Since PI is not able to penetrate intact membranes of living cells, the cells with dramatically increased fluorescent intensity in the emission filter of 610 nm should not be considered and be excluded from the count.

In this series of assays, each sample was measured at a flow rate of 50 μL/min for 30 s. During the cell count, the number of cells in the control group was taken as 100%. Based on the obtained results, we determined no observed effect concentrations (NOEC) and calculated the effective concentrations of the CNMs caused 10% (EC_10_) and 50% (EC_50_) inhibition of microalgae growth rate after 96 h and 7 days of exposure. The calculation of EC_10_ and EC_50_ values was performed by nonlinear regression fit in GraphPad Prism 8.0.2 (GraphPad Software, San Diego, CA, USA). The 96 h and 7 days EC_10_ and EC_50_ descriptors were chosen as one of the most common values used for evaluation of cytotoxic effects in macroalgae bioassay [94,95].

To determine the size of microalgae cells, a size calibration kit, batch F13838 (Molecular Probes, Eugene, OR, USA) with the certified size distribution of 1, 2, 4, 6, 10, and 15 μm was used for the forward scatter emission channel. The distribution of control group cells between the size ranges were taken as 100%.

The change in esterase activity of microalgae exposed to the CNMs was evaluated using non-fluorescent lipophilic dye fluorescein diacetate (FDA), which activates under the influence of microalgae esterases [96]. Thus, the changes in metabolic activity of microalgae cells can be evaluated according to the intensity of fluorescein fluorescence inside the cells, registered in the emission filter of 525 nm.

The membrane potential of microalgae cells was assessed by a lipophilic, positively charged fluorescent dye 3,3′-dihexyloxacarbocyanine iodide (DiOC_6_), which is capable of binding to membranes (mitochondria and endoplasmic reticulum) and other hydrophobic negatively charged cell structures [97]. If the inner membrane potential of the cell decreases and the cells become more electronegative compared to control, then more of the dye will be absorbed, which indicates hyperpolarization. If the membrane potential increases and the cell becomes less electronegative compared to the control group, the dye will be removed from the cell and thus indicate depolarization [98].

The level of reactive oxygen species (ROS) generation in microalgae cells exposed to the nanoparticles was assessed using non-fluorescent dye 2′,7′-dichlorodihydrofluorescein diacetate (H_2_DCFDA) which activates in the presence of ROS and indicates general oxidative stress in microalgae cells [99]. The efficiency of the staining was furthermore checked by the test with the addition of 1.7 µM hydrogen peroxide, which increased the MFI of the cells in order of magnitude.

In the cases of esterase activity, membrane potential, and ROS generation changes, the flow cytometry measurements were performed at a flow rate of 100 μL/min until 2000 cells were registered to adequately compare cellular responses in each treatment condition. In this series of assays, the MFI of the control group cells in a 525 nm filter was taken as 100% and considered as a positive control. To obtain a negative control level, microalgae cells were heat treated at 98 °C for 15 min, cooled at room temperature, and measured the same way as the other samples in this series (in quadruplicate). The MFI of the negative control group was taken as 0%. All the obtained results were normalized using the data of control groups. Based on the obtained results, we determined NOEC concentrations for each endpoint and compared the cellular responses to different treatment conditions. The 3 and 24 h registration points allow the detection of possible early metabolic response of the algae over short exposure periods and dynamic change in that response, respectfully [100,101].

### 4.5. Microscopy

Morphological changes of microalgae cells were observed and captured by an optical microscope Axio Observer A1 (Carl Zeiss, Oberkochen, Germany).

### 4.6. FTIR and Raman Spectroscopy

The samples obtained, as described in Section 4.3, were analyzed by IRTracer-100 FTIR spectrometer (Shimadzu, Kyoto Japan) with a DTGS detector. In total, 100 scans with a wide spectral range from 400 to 4000 cm^−1^ were collected to obtain the appropriate signal-to-noise ratio. The spectral resolution was set to 4 cm^−1^. The prepared samples were mixed with 300 mg of potassium bromide powder (KBr) at a concentration of 0.03 wt%. The mixture was pressed in a pellet for 10 min at 10 tons (Ø 13 mm).

Raman spectra were collected with an NTEGRA II Raman microscope and spectrometer (NT-MDT Spectrum Instruments, Moscow, Russia) equipped with a 473 nm laser and a 100-fold objective. The signal accumulation time for each measurement was 30 s. The spectra were analyzed by a grating-type spectrometer (diffraction grating 1800 lines/mm) with an electrically cooled CCD camera.

### 4.7. Statistical Analysis

Statistical analyzes were performed by GraphPad Prism 8.0.2 (GraphPad Software, San Diego, CA, USA). The statistical significance was tested by one-way ANOVA with Dunnett’s multiple comparisons tests. The normality of residuals was checked by the Anderson–Darling test. A value of *p* ≤ 0.05 was considered statistically significant.

## 5. Conclusions

In general, this study demonstrated the level of toxicity and mode of toxic action of multiwalled carbon nanotubes (CNTs), fullerene (C_60_), graphene powder (Gr), and graphene oxide (GrO) in microalgae *H.akashiwo*. The toxic impact of the used CNMs on microalgae growth rate reduces in the following order: CNTs > GrO > Gr > C_60_. Oxidative stress and membrane depolarization was indicated among the main toxic action of CNTs and GrO. At the same time, Gr and C_60_ reduced the toxic action with time and had no negative impact on microalgae even at a concentration of 125 mg/L. Moreover, this study revealed the presence of structural deformation in C_60_ and Gr after seven days of contact with microalgae cells. Therefore, the results of the biotransformation assay correlate with the toxic properties of the CNMs, and less toxic materials are more likely to be transformed after contact with aquatic organisms.

## Figures and Tables

**Table 1:** The toxicity descriptors of CNMs exposure to *H. akashiwo*.

Duration of Exposure	Toxicity Descriptor	CNTs, mg/L	C_60_, mg/L	Gr, mg/L	GrO, mg/L
Growth Rate Inhibition					
96 h	NOEC	1	25	5	5
	EC_10_	1.52 (0.99–2.25)	34.57 (9.54–63.90)	13.80 (8.52–20.51)	8.22 (4.84–12.90)
	EC_50_	18.98 (15.92–22.49)	414.0 (218.10–1431)	159.40 (137.10–192.60)	76.77 (66.82–87.68)
7 days	NOEC	1	1	1	1
	EC_10_	1.37 (0.98–1.89)	n/a	n/a	34.39 (24.69–44.08)
	EC_50_	13.41 (11.52–15.53)	n/a	n/a	118.0 (109.30–129.10)
Cell size change					
96 h	NOEC	10	10	25	25
7 days	NOEC	50	25	25	10

95% confidence limits presented in the parentheses; n/a, the effect was not observed even at the highest concentrations of the sample.

**Table 2:** The no observed effect concentrations (NOEC) of esterase activity, membrane potential, and ROS generation changes in *H. akashiwo* cells after the exposure to CNMs.

Endpoint	Duration of Exposure	CNTs, mg/L	C_60_, mg/L	Gr, mg/L	GrO, mg/L
Esterase activity change	3 h	25	25	50	25
	24 h	50	25	50	50
Membrane potential change	3 h	<1	50	10	10
	24 h	<1	50	50	25
ROS generation change	3 h	25	25	50	50
	24 h	25	10	50	10

**Table 3:** Characteristics of the used carbon nanomaterials.

Sample	Size	Purity	Synthesis or Manufacturer Information
CNTs	Diameter: 6–13 nm; Length: 2.5–20 µm	>98% (Trace metals—13,567 mg/kg, including Al—10,000 mg/kg, Co—2652 mg/kg)	Batch Number: MKCM1457; Sigma Aldrich, St. Louis, MO, USA
C_60_	Diameter: 0.8 nm	>95.5% (oxide C_60_)	Batch Number: 120722; Modern Synthesis Technology (MST), Saint-Petersburg, Russia
Gr	Thickness: 3–10 nm; Diameter: 0.5–10 µm	>99%	Type #1, CAS#: 1034343-98-0; Modern Synthesis Technology (MST), Saint-Petersburg, Russia
GrO	Diameter:10–100 µm	Carbon: 46%; Oxygen: 49%; Hydrogen: 2.5%; Sulfur: 2.5%	CAS#: 1034343-98-0; Modern Synthesis Technology (MST), Saint-Petersburg, Russia

**Table 4:** Bioassay endpoints and registration parameters.

Endpoint	Fluorescent Dye or Registered Parameter	Duration of Microalgae Exposure before the Measurement	Dye Concentration/Duration of Staining	Emission Channel/Band Width, nm
Growth rate inhibition	PI	96 h, 7 days	15 µM/20 min	610/20
Cell size change	Forward scatter intensity (size calibration kit F13838 by Molecular Probes, Eugene, OR, USA)	96 h, 7 days	–	FSC
Esterase activity change	FDA	3, 24 h	50 µM/20 min	525/40
Membrane potential change	DiOC_6_	3, 24 h	0.5 µM/20 min	525/40
ROS generation change	H_2_DCFDA	3, 24 h	50 µM/30 min	525/40

ROS, Reactive oxygen species; PI, Propidium iodide; FDA, Fluorescein diacetate; DiOC_6_, 3,3′-dihexyloxacarbocyanine iodide; H_2_DCFDA, 2′,7′-dichlorodihydrofluorescein diacetate; FSC, the emission channel indicating forward scatter of blue laser 488 nm.

## Data Availability

Not applicable.

## Supplementary Materials

The following supporting information can be downloaded at: https://www.mdpi.com/article/10.3390/ijms241210020/s1.
