# Supplementary material for: Toxicity and Biotransformation of Carbon-Based Nanomaterials in Marine Microalgae Heterosigma akashiwo
Source: Int J Mol Sci. 2023 Jun 12;24(12):10020. doi: 10.3390/ijms241210020 (PMC10297852; doi:10.3390/ijms241210020)
Supplement: Supplementary file 1 [file ijms-24-10020-s001.zip › ijms-2416845-supplementary.pdf]

Toxicity and Biotransformation of Carbon-Based Nanomaterials in Marine Microalgae *Heterosigma akashiwo*

Konstantin Pikula [1], Seyed Ali Johari [2], Ralph Santos-Oliveira [3,4] and Kirill Golokhvast [1,5,*]

[1] Polytechnical Institute, Far Eastern Federal University, 10 Ajax Bay, Russky Island, 690922 Vladivostok, Russia
[2] Department of Fisheries, Faculty of Natural Resources, University of Kurdistan, Pasdaran St, Sanandaj 66177-15175, Iran
[3] Laboratory of Nanoradiopharmaceuticals and Synthesis of Novel Radiopharmaceuticals, Nuclear Engineering Institute, Brazilian Nuclear Energy Commission, Rua Hélio de Almeida 75, Rio de Janeiro 21941906, Brazil
[4] Laboratory of Nanoradiopharmaceuticals and Radiopharmacy, Rio de Janeiro State University, R. São Francisco Xavier, 524, Rio de Janeiro 23070200, Brazil
[5] Siberian Federal Scientific Center of Agrobiotechnology RAS, Centralnaya Str., Presidium, 633501 Krasnoobsk, Russia
* Correspondence: golokhvast@sfsca.ru

Table S1. The statistical significance calculation of growth rate inhibition, cell size change, esterase activity, membrane potential, and ROS generation in *H. akashiwo* cells.

Concentration, mg/L	CNTs	C60	Gr	GrO
**Growth rate inhibition, 96 h**				
1	0.8565 (ns)	0.9994 (ns)	0.9465 (ns)	0.0822 (ns)
5	<0.0001 (****)	0.1846 (ns)	0.9968 (ns)	0.4746 (ns)
10	<0.0001 (****)	0.0640 (ns)	0.0170 (*)	<0.0001 (****)
25	<0.0001 (****)	0.0936 (ns)	<0.0001 (****)	<0.0001 (****)
50	<0.0001 (****)	0.0345 (*)	<0.0001 (****)	<0.0001 (****)
75	<0.0001 (****)	<0.0001 (****)	<0.0001 (****)	<0.0001 (****)
100	<0.0001 (****)	<0.0001 (****)	<0.0001 (****)	<0.0001 (****)
125	<0.0001 (****)	<0.0001 (****)	<0.0001 (****)	<0.0001 (****)
**Growth rate inhibition, 7 days**				
1	0.0813 (ns)	0.9995 (ns)	0.1519 (ns)	0.0852 (ns)
5	<0.0001 (****)	0.0264 (*)	0.0235 (*)	0.0212 (*)
10	<0.0001 (****)	0.0148 (*)	0.0002 (***)	0.0003 (***)
25	<0.0001 (****)	0.0088 (**)	<0.0001 (****)	<0.0001 (****)
50	<0.0001 (****)	0.0647 (ns)	<0.0001 (****)	<0.0001 (****)
75	<0.0001 (****)	0.0578 (ns)	0.9184 (ns)	<0.0001 (****)
100	<0.0001 (****)	0.9067 (ns)	0.0009 (***)	<0.0001 (****)
125	<0.0001 (****)	0.7878 (ns)	<0.0001 (****)	<0.0001 (****)
**Cell size change, 96 h**				
10	0.0628 (ns)	0.2806 (ns)	0.6840 (ns)	0.2813 (ns)
25	<0.0001 (****)	0.0018 (**)	0.2774 (ns)	0.1005 (ns)
50	<0.0001 (****)	<0.0001 (****)	0.0435 (*)	0.0003 (***)
75	<0.0001 (****)	<0.0001 (****)	0.0016 (**)	0.9837 (ns)
100	<0.0001 (****)	<0.0001 (****)	<0.0001 (****)	0.0012 (**)
125	<0.0001 (****)	<0.0001 (****)	<0.0001 (****)	<0.0001 (****)
Cell size change, 7 days				

Concentration, mg/L	CNTs	C60	Gr	GrO
10	0.8743 (ns)	0.7312 (ns)	0.1551 (ns)	0.0815 (ns)
25	0.9353 (ns)	0.0943 (ns)	0.3101 (ns)	<0.0001 (****)
50	0.9763 (ns)	0.0068 (**)	0.0114 (*)	<0.0001 (****)
75	0.0038 (**)	0.0302 (*)	<0.0001 (****)	<0.0001 (****)
100	<0.0001 (****)	0.0077 (**)	<0.0001 (****)	0.0040 (**)
125	<0.0001 (****)	0.0004 (***)	<0.0001 (****)	0.5229 (ns)
**Esterase activity change, 3 h**				
1	0.9777 (ns)	0.8151 (ns)	0.9728 (ns)	0.9997 (ns)
10	0.2180 (ns)	>0.9999 (ns)	0.9946 (ns)	0.9867 (ns)
25	0.9667 (ns)	0.4053 (ns)	0.4241 (ns)	0.4888 (ns)
50	0.0002 (***)	0.0013 (**)	0.6939 (ns)	<0.0001 (****)
**Esterase activity change, 24 h**				
1	0.9792 (ns)	0.1263 (ns)	0.2951 (ns)	0.9999 (ns)
10	0.9557 (ns)	0.7828 (ns)	0.1628 (ns)	0.9851 (ns)
25	0.6532 (ns)	0.7801 (ns)	0.1140 (ns)	0.9999 (ns)
50	0.8160 (ns)	0.0005 (**)	0.1652 (ns)	0.5621 (ns)
**Membrane potential change, 3 h**				
1	<0.0001 (****)	0.6373 (ns)	0.1838 (ns)	0.1657 (ns)
10	<0.0001 (****)	0.6209 (ns)	0.0881 (ns)	0.1049 (ns)
25	<0.0001 (****)	0.3499 (ns)	0.0107 (*)	0.0074 (**)
50	<0.0001 (****)	0.3301 (ns)	0.1120 (ns)	0.0136 (*)
**Membrane potential change, 24 h**				
1	0.0006 (***)	0.9835 (ns)	0.8623 (ns)	0.9569 (ns)
10	<0.0001 (****)	0.1924 (ns)	0.0860 (ns)	0.9993 (ns)
25	<0.0001 (****)	0.3057 (ns)	0.1285 (ns)	0.9327 (ns)
50	<0.0001 (****)	0.0581 (ns)	0.4097 (ns)	<0.0001 (****)
**ROS generation change, 3 h**				
1	0.6905 (ns)	>0.9999 (ns)	0.9549 (ns)	0.9419 (ns)
10	0.9960 (ns)	0.9922 (ns)	0.6762 (ns)	0.8420 (ns)
25	>0.9999 (ns)	0.7576 (ns)	0.6435 (ns)	0.9999 (ns)
50	0.0009 (***)	0.0268 (*)	0.9989 (ns)	0.6460 (ns)
**ROS generation change, 24 h**				
1	0.2073 (ns)	0.1793 (ns)	0.5034 (ns)	0.1789 (ns)
10	0.3285 (ns)	0.2060 (ns)	0.2365 (ns)	0.1127 (ns)
25	0.9943 (ns)	0.0018 (**)	0.2885 (ns)	0.0014 (**)
50	<0.0001 (****)	0.0221 (*)	0.6827 (ns)	<0.0001 (****)

ROS, Reactive oxygen species; ns, nonsignificant ($p > 0.05$); *, $p < 0.05$; **, $p < 0.005$; ***, $p < 0.0005$; ****, $p < 0.0001$.
